# Rare case of neglected large sacral Chordoma in a young female treated by wide En bloc resection and Sacrectomy

**DOI:** 10.1186/s12885-018-5012-3

**Published:** 2018-11-14

**Authors:** Zi Hao Phang, Xue Yi Saw, Noreen Fadzlina Binti Mat Nor, Zolqarnain Bin Ahmad, Sa’adon Bin Ibrahim

**Affiliations:** 1 Department of Orthopaedics, Hospital Sultan Ismail, Johor Bahru, Malaysia; 2 University Malaya, Kuala Lumpur, Malaysia

**Keywords:** Neglected, Sacral Chordoma, Tumour, En bloc resection, Sacrectomy, Oncology

## Abstract

**Background:**

Sacral chordoma is a locally aggressive malignant tumour originating from ectopic notochordal cells. The natural history of sacral chordoma is a slow growing tumour arising at the midline of the lower sacrum that can invade the sacrum and progressively increase in size expanding cranially and anteriorly. Metastasis is very rare even when the tumour is large. Sacral chordoma affects males more than females and is more commonly found in middle age and elderly patients.

**Case presentation:**

A 25 years old female had neglected an extremely large midline sacral mass for 2 years. On presentation to hospital, she had been bed bound for the past 2 years. The sacral mass was so large that it prevented her from lying down supine and sitting on the wheelchair comfortably. Clinical examination showed a 40 cm × 30 cm × 20 cm hard mass over the sacrum that involved both buttocks and the gluteal fold. Neurological exam of bilateral lower limb was normal. Computed Tomography Scan of the Pelvis showed a large destructive sacrococcygeal mass measuring 43 cm × 38 cm × 27 cm with extension into the presacral space resulting in anterior displacement of the rectum, urinary bladder and uterus; and posterior extension into the dorsal soft tissue with involvement of the gluteus, piriformis, and left erector spinae muscles. Biopsy taken confirmed Chordoma. This patient was managed by a multidisciplinary team in an Oncology referral centre. The patient had undergone Wide En Bloc Resection and Sacrectomy, a complex surgery that was associated with complications namely bleeding, surgical site infection and neurogenic bowel and bladder. Six months post operatively the patient was able to lie supine and sit on wheelchair comfortably. She required extensive rehabilitation to help her ambulate in future.

**Conclusion:**

This is a rare case of neglected sacral chordoma in a young female treated with Wide En Bloc Resection and Sacrectomy associated with complications of this complex surgery. Nevertheless, surgery is still worthwhile to improve the quality of life and to prevent complications secondary to prolonged immobilization. A multidisciplinary approach is ideal and team members need to be prepared to address the complications once they arise.

## Background

Sacral tumour is a rare clinical entity [1]. The most common type of primary sacral tumour is chordoma [1, 2]. Sacral chordoma is a locally aggressive malignant tumour originating from ectopic notochordal cells [1]. Chordoma predominantly affects the axial skeleton for example the sacrum, skull and other mobile segments of spine [1]. The natural history of sacral chordoma is a slow growing tumour arising at the midline of the lower sacrum that can locally invade the sacral bone and progressively increase in size expanding cranially and anteriorly [1]. This sacral tumour if large enough can push abdominal and pelvic organs anteriorly [1]. Metastasis is very rare even when the tumour is very large [1]. In addition, the tumour rarely invades the rectum or genitourinary organs because it is confined by the thick presacral fascia and periosteum [1]. Sacral chordoma affects males more than females and is more commonly found in middle age and elderly patients [1]. The current trend of management of sacral chordoma is En Bloc Resection and Sacrectomy to excise the whole tumour together with adequate surgical margin to reduce the risk of local recurrence [1–5]. This is a complex surgery which involves a multidisciplinary team and has many potential complications such as wound dehiscence, neurogenic bowel and bladder, massive haemorrhage and sacropelvic instability [6–11].

Neglected case of Sacral Chordoma is rarely seen. This case report described approach to surgical treatment and outcome of a case of sacral chordoma in an uncommon age group (young adulthood) that had been neglected for 2 years by a patient from a disadvantaged socioeconomic background. It allowed a description of the natural history of chordoma if left untreated, which confirmed what had been described theoretically.

## Case presentation

This is a case of a 25 years old Malay girl with learning disability and no significant past medical history, who started noticing a sacral mass since August 2015. The mass was painless and gradually increasing in size. The family members of this patient brought her to a traditional healer. They did not seek any medical treatment until late 2017. By this time, the mass over the sacrum was extremely large. Family members claimed the mass was preventing the patient from lying down flat supine. The patient was also unable to ambulate for the past 2 years. Hence, she was bedbound most of the time. It was difficult for her to sit on the wheelchair. She also felt tired to move because the mass was quite heavy. The family members claimed when the patient was lying down flat, she had to flex her hips and knees to achieve a more comfortable position. In addition, she often slept either in prone position or in supine with multiple pillows below her body. The mother also claimed over the last 2 months, the patient’s body had been getting thinner despite her physical weight was increasing due to the increase in size of the sacral mass. The patient had been passing stool and urine in pampers. There was no past medical history and no family history of cancer. Socially, the patient lived with her mother and siblings. The mother was the main care taker. Her father passed away 10 years ago because of heart attack. The patient previously attended a special needs school, but she stopped going to school since 2015 after developing the sacral mass.

**Fig. 1:**
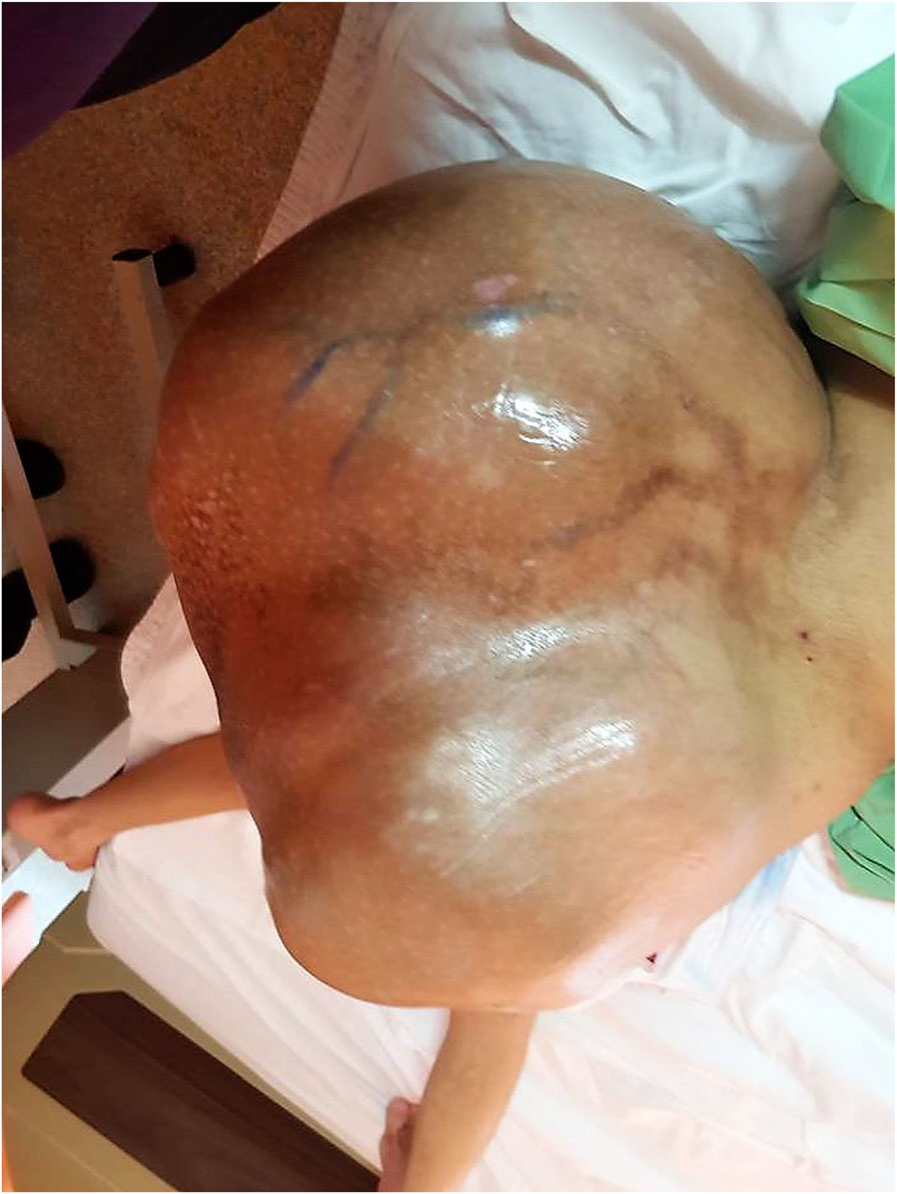
Preoperative clinical photograph of sacral mass

This patient was managed in the Southern Region referral centre for Orthopaedic Oncology in Malaysia. On clinical examination in the Orthopaedic Oncology ward, the patient appeared cachexic, she had slightly pale conjunctiva, but she was not dysmorphic. Vital signs were Blood Pressure 142/90, Pulse Rate 98 beats per minute and Temperature 37 degrees Celsius. There was a large mass 40 cm × 30 cm × 20 cm over the sacrum. The mass was firm to hard in consistency and involved both buttocks and the gluteal fold (Fig. 1). Dilated veins were noted under the skin overlying the sacral mass. Neurological exam of bilateral lower limb was normal. However, there was generalized wasting of all muscles over the bilateral lower limb. Anal tone was intact.

**Fig. 2:**
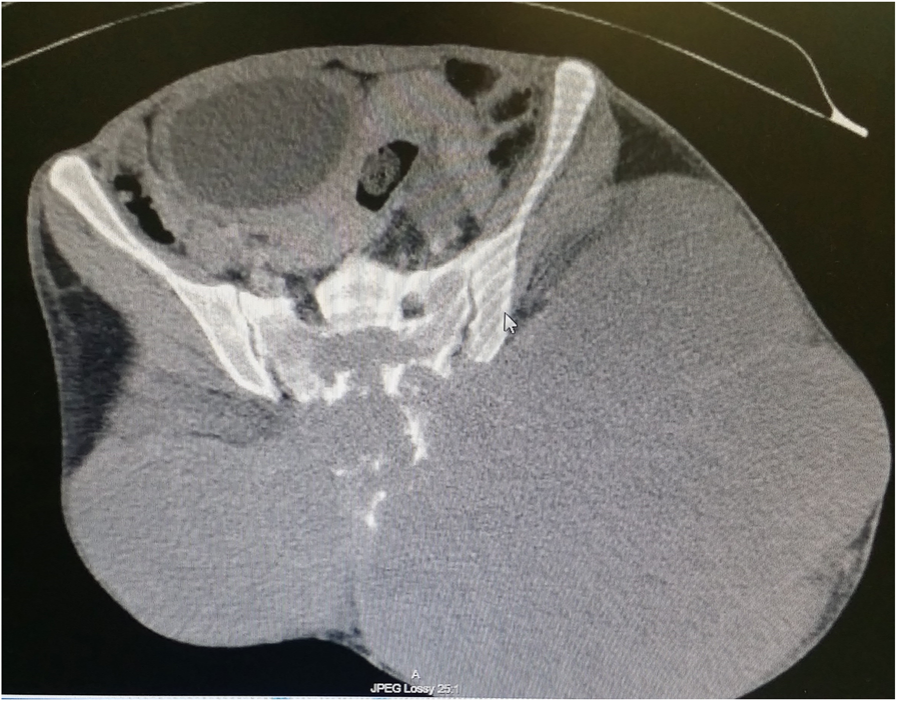
Coronal view Computed Tomography Scan of Pelvis

**Fig. 3:**
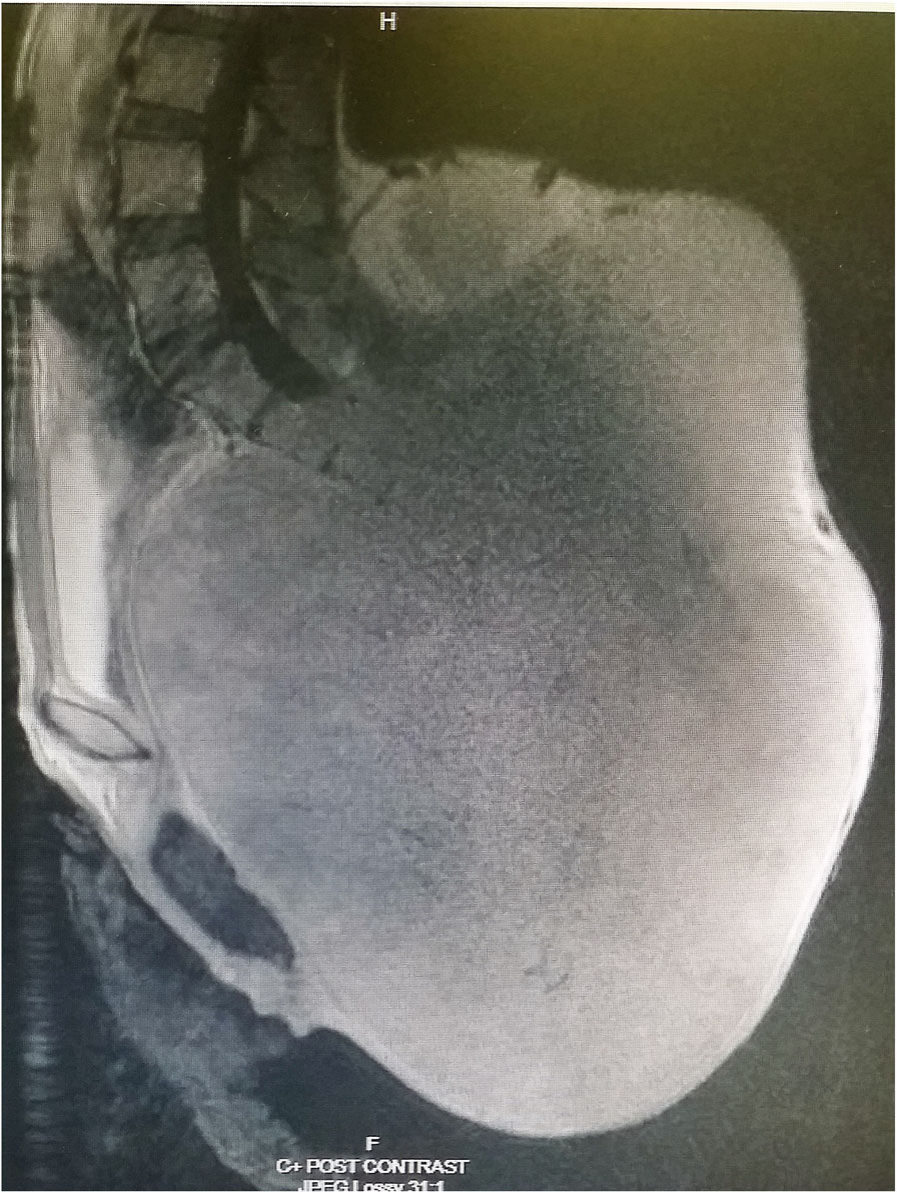
Sagittal view Computed Tomography Scan of Pelvis

**Fig. 4:**
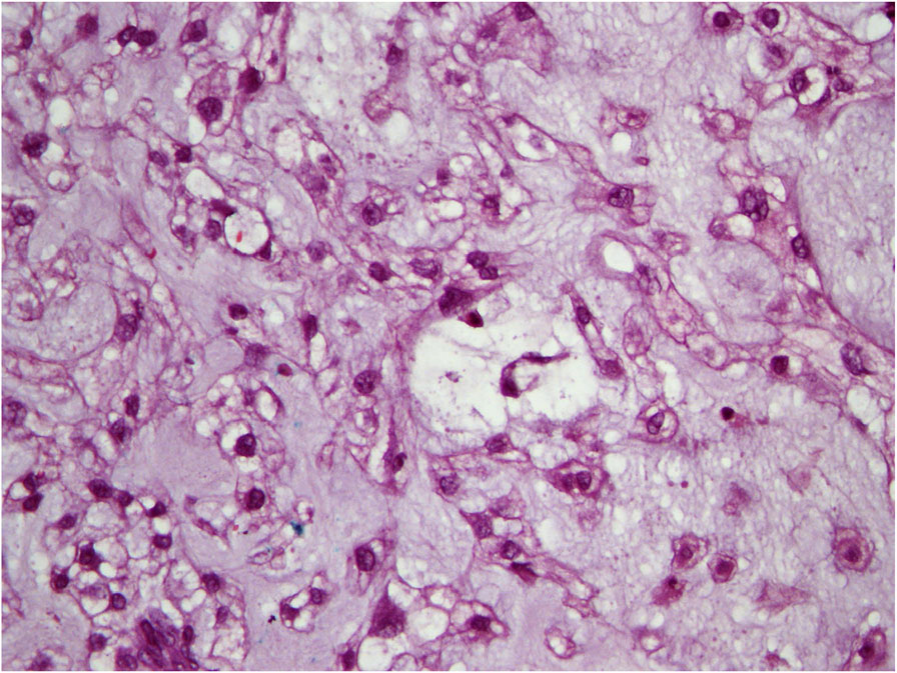
Histopathological Morphology of Tumour

Laboratory investigations taken were unremarkable. Computed Tomography of the Pelvis showed a large destructive sacrococcygeal mass measuring 43 cm × 38 cm × 27 cm with extension into the presacral space resulting in anterior displacement of the rectum, urinary bladder and uterus and posterior extension into the dorsal soft tissue with involvement of the gluteus, piriformis, and left erector spinae muscles (Figs. 2 and 3). Superior margin of the sacral bone involvement was up to S2. The mass was predominantly of fluid density with internal enhancing septation and calcifications which suggested primary chordoma more likely (Figs. 2 and 3). Magnetic Resonance Imaging done showed similar findings. Skeletal Survey Radiograph did not show any distant metastasis. A Trucut biopsy of the mass was done. Histopathological analysis showed tumour cells with “physaliphorous cells” positive for pancytokeratin, EMA, Vimentin and S-100 immunohistochemistry stainings with minimal mitotic figures and mild nuclear pleomorphism (Fig. 4). Brachyury immunohistochemistry staining was not available in our centre. However, the clinical history, morphology of tumour on microscopy and immunohistochemistry staining available were consistent sacral chordoma.

The diagnosis of Sacral Chordoma was confirmed. Multidisciplinary team discussion done among Orthopaedic Oncology, General Surgery, Obstetrics and Gynaecology, Blood Bank, Anaesthetic and Plastic Surgery teams. A family conference was done. The family’s aim was for removal of the sacral mass to allow the patient lie supine on bed and sit on the wheelchair.

**Fig. 5:**
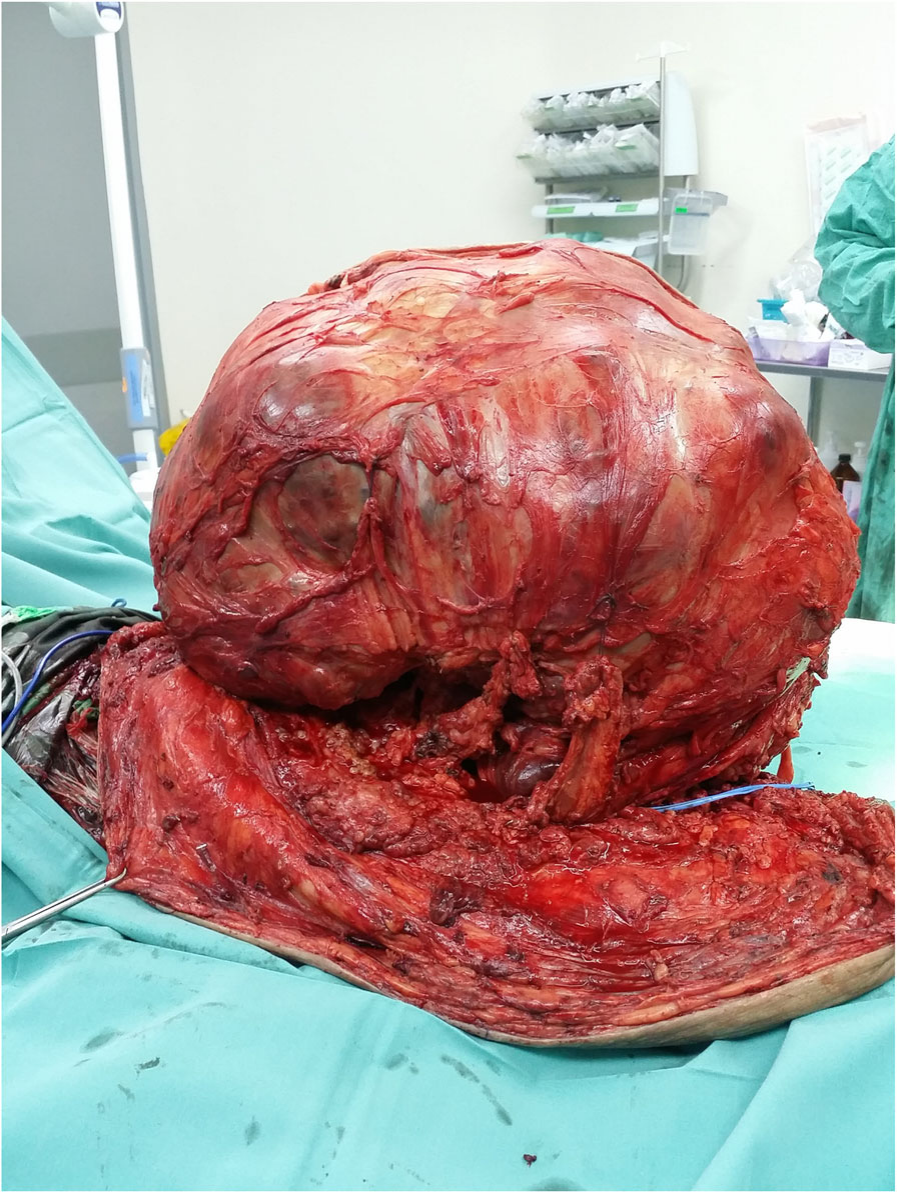
Intraoperative photograph for sacral mass 1

**Fig. 6:**
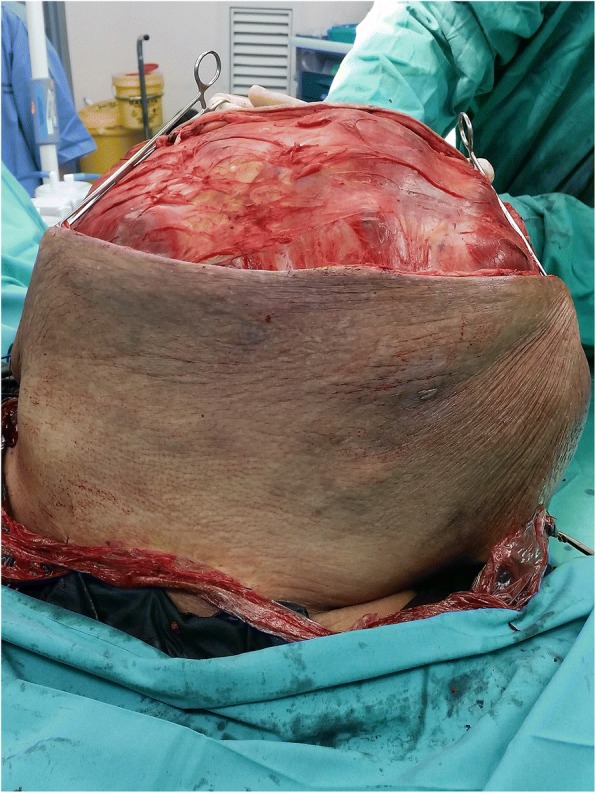
Intraoperative photograph for sacral mass 2

**Fig. 7:**
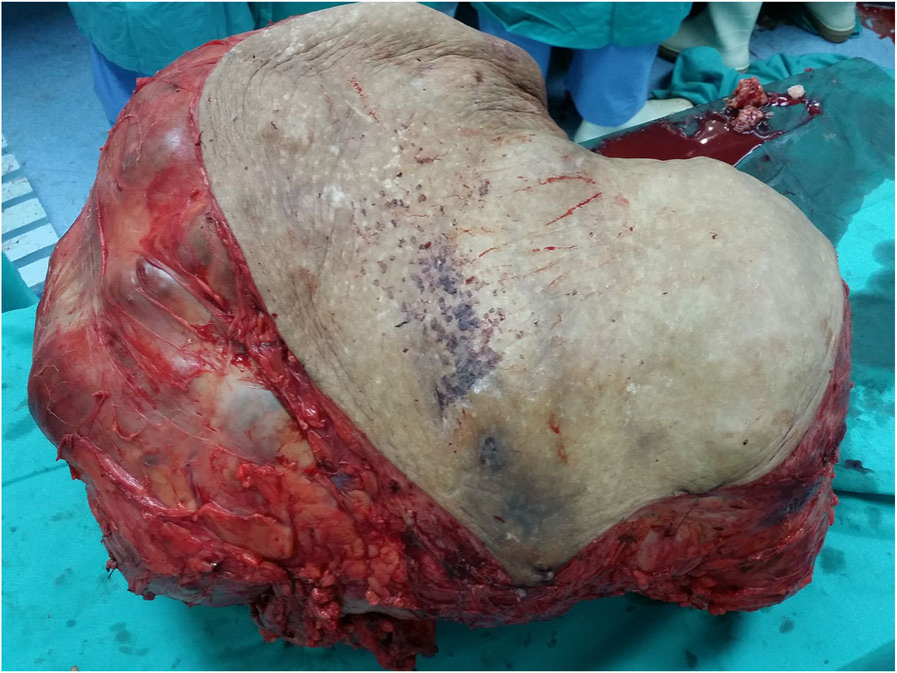
Intraoperative photograph for sacral mass 3

Subsequently, the patient undergone Wide Resection and En Bloc Sacrectomy. The Posterior-Only Approach was used with a “Mercedes Star” 3 limbed incision. Duration of surgery was 8 h. The patient was supported with blood products transfusion during surgery. Intraoperatively, the sacral tumour had eroded the sacral bone from S2 to S5 (Figs. 4, 5 and 6). Sacrectomy was done at the level of S2. Sacral nerve roots S2-S5 were all infiltrated by the mass and therefore were unable to be preserved. The mass and surrounding gluteal muscles invaded by the tumour were also all resected. All resection margins were less than 1 mm from the tumour. Primary closure was done without any distant or local flap as per consultation with Plastic Surgery team. The tumour weight was 25 kg (Figs. 5, 6 and 7). Post operatively, the patient was monitored in Intensive Care Unit for 3 days. The patient developed neurogenic bowel and bladder post sacrectomy requiring enema and long-term urinary catheter. In addition, the post-operative course was complicated by wound breakdown and surgical site infection requiring wound debridement. Dressing was done as per local protocol until wound bed granulating well. Split Skin Graft was done about 3 months post wide resection once the tissue culture results were free of significant infection.

**Fig. 8:**
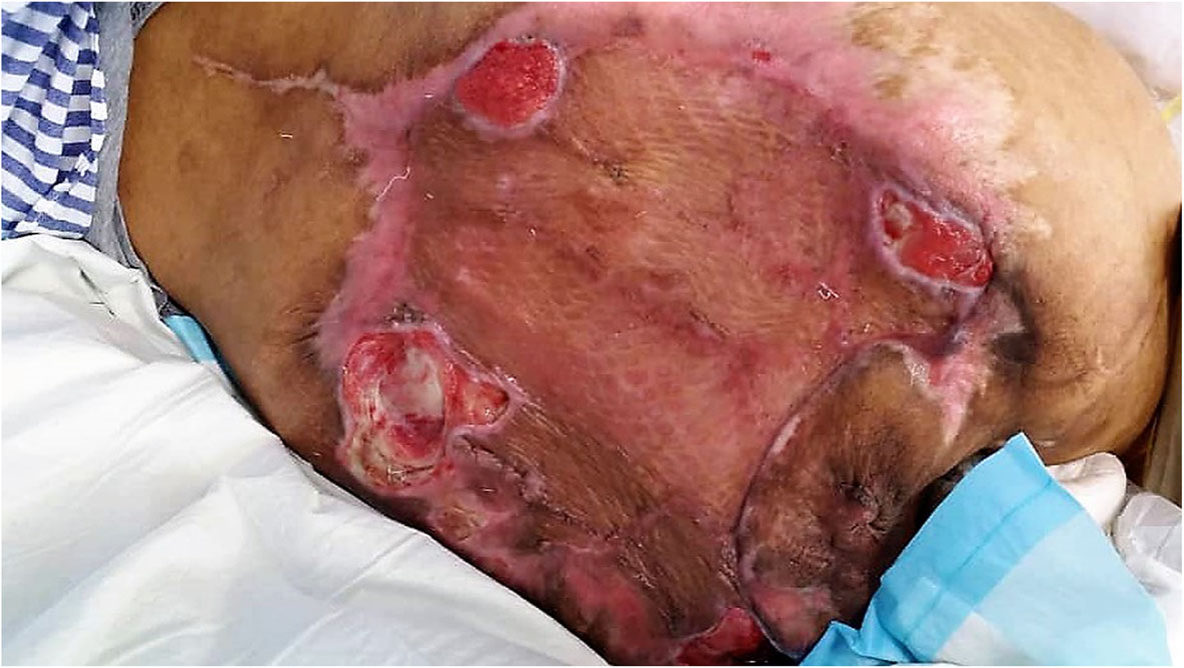
Clinical photograph 5 months post resection

The patient also required extensive rehabilitation for transfer, ambulation and bowel and bladder care. Rehabilitation was difficult because the patient had learning disability and she had been habitually keeping her hips and knees flexed because of the sacral tumour for the past 2 years. During the last review 5 months post operatively, patient was able to sit on the wheelchair comfortably. The surgical wound was healing well with good uptake of the Split Skin Graft (Fig. 8).

## Discussion and conclusion

Sacral chordoma is a rare, slow growing, locally aggressive malignant tumour that rarely metastasize even if it presents late [1]. This had been shown to be true in this case study of a neglected sacral chordoma which presented with a 25 kg tumour without any evidence of distant metastasis. In addition, there were no invasion of the adjacent colorectal, genitourinary and gynaecological organs. This is one of the malignant tumours that lack many features of malignancy on histopathological analysis such as mitosis, anaplasia and increase nuclear-cytoplasmic ratio. Most sacral chordoma occurred in middle age or elderly adults [1]. The patient in this case study was a rare case of sacral chordoma in a young female.

The management of Sacral Chordoma is challenging and associated with many complications and morbidities [10]. Patients with Sacral Chordoma are best managed in an Oncology Hospital using a Multidisciplinary approach [5]. The primary treatment of Sacral Chordoma is surgery [1]. En Bloc Resection with adequate surgical margin and Sacrectomy offered the best chance of reducing local recurrence [1, 3, 4, 6, 11]. Radiotherapy and Chemotherapy are rarely effective [1]. Complications of Sacrectomy remain prevalent despite advancement in surgical techniques and equipment [8]. Most commonly described complications in the literature were massive haemorrhage, wound complications (wound dehiscence, surgical site infection), neurological deficits (neurogenic bowel and bladder, sciatic nerve or lumbosacral plexus injury) and instrumentation problems (related to sacropelvic fixation) [7–11]. The patient in this case study had three out of four complications mentioned above namely haemorrhage, surgical site infection and neurogenic bowel and bladder. Firstly, this patient had many risk factors for haemorrhage as described by Tang and colleagues [8]. There was no expertise for cross aortic clamping or intraaortic balloon at our centre. The patient was admitted preoperatively for intravenous iron transfusion and maintained adequate blood transfusion perioperatively to maintain patient’s haemodynamics. Secondly, the patient also had many risk factors for surgical site infection as described by Sciubba and colleagues [7]. The patient was quite cachexic because of the large tumour that a vertical rectus abdominus muscle flap was not possible. After consultation with plastic surgery, the only option was for primary closure of the wound using the remaining skin flap. Nevertheless, this was complicated by wound breakdown and surgical site infection. Subsequently, local wound dressing was done to prepare the wound bed. Split skin graft was done to cover the wound once tissue sample was clear of significant infection. There was good uptake of the split skin graft and the wound healed well. Thirdly, the tumour for so large that it infiltrated nerve roots of S2 to S5 bilaterally. Therefore, decision was made to sacrifice the involved sacral nerve roots. The complication of neurogenic bowel and bladder was expected and this concurred with the findings of Todd and colleagues who concluded inability to preserve at least one S3 sacral nerve root had high risk of neurogenic bowel and bladder [9]. We were also unable to obtain adequate resection margin given the extremely large size of this tumour.

Despite all the complications, surgical resection of this neglected sacral chordoma was worthwhile to prevent the complications for immobility and bedridden caused by this large, heavy and bulky tumour. The aims after surgery were for intensive rehabilitation to mobilize the patient and to monitor closely for local recurrence of the tumour.

In conclusion, this is a rare case of neglected sacral chordoma in a young female treated with Wide En Bloc Resection and Sacrectomy. There are many complications associated with this complex surgery. Nevertheless, surgery is still worthwhile to improve the quality of life, to prevent complications secondary to prolonged immobilization and bedridden. A multidisciplinary approach is required for such complex case and all team members need to be prepared to address the complications once they arise.
